# Characterization of the complete mitochondrial genome sequences of three Merulinidae corals and novel insights into the phylogenetics

**DOI:** 10.7717/peerj.8455

**Published:** 2020-01-24

**Authors:** Wentao Niu, Jiaguang Xiao, Peng Tian, Shuangen Yu, Feng Guo, Jianjia Wang, Dingyong Huang

**Affiliations:** Laboratory of Marine Biology and Ecology, Third Institute of Oceanography, Ministry of Natural Resources, Xiamen, Fujian, China

**Keywords:** *Dipsastraea rotumana*, *Favites pentagona*, *Hydnophora exesa*, Scleractinia, Mitochondrial genome, Characterization, Phylogenetic analysis

## Abstract

Over the past few decades, modern coral taxonomy, combining morphology and molecular sequence data, has resolved many long-standing questions about scleractinian corals. In this study, we sequenced the complete mitochondrial genomes of three Merulinidae corals (*Dipsastraea rotumana*, *Favites pentagona*, and *Hydnophora exesa*) for the first time using next-generation sequencing. The obtained mitogenome sequences ranged from 16,466 bp (*D. rotumana*) to 18,006 bp (*F. pentagona*) in length, and included 13 unique protein-coding genes (PCGs), two transfer RNA genes, and two ribosomal RNA genes . Gene arrangement, nucleotide composition, and nucleotide bias of the three Merulinidae corals were canonically identical to each other and consistent with other scleractinian corals. We performed a Bayesian phylogenetic reconstruction based on 13 protein-coding sequences of 86 Scleractinia species. The results showed that the family Merulinidae was conventionally nested within the robust branch, with *H. exesa* clustered closely with *F. pentagona* and *D. rotumana* clustered closely with *Favites abdita*. This study provides novel insight into the phylogenetics of species within the family Merulinidae and the evolutionary relationships among different Scleractinia genera.

## Introduction

Merulinidae (Verrill, 1865) is a clade of corals that belongs to the order Scleractinia and currently comprises 149 species across 24 genera (Huang et al., 2014a; see also WoRMS Editorial Board, 2019). These species are mainly distributed in the Indo-Pacific and Caribbean regions, but are absent in the eastern Pacific. Initially, Verrill (1865) posited that the family Merulinidae (type genus *Merulina*) fell within the suborder Fungacea. Merulinidae, however, was not recognized as a valid family by subsequent authors (Quenstedt, 1881; Quelch, 1886; Vaughan, 1918; Hoffmeister, 1925; Faustino, 1927; Matthai, 1928; Yabe, Sugiyama & Eguchi, 1936), until Vaughan & Wells (1943) revived it. Some modifications were proposed successively (Umbgrove, 1940; Chevalier, 1975; Veron, 1985). Nested within the ‘Bigmessidae’ (Budd, 2009), Merulinidae was polyphyletic and its species belonged to multiple divergent subclades (Fukami et al., 2004; Fukami et al., 2008; Huang et al., 2009; Huang et al., 2011; Benzoni et al., 2011; Budd & Stolarski, 2011; Arrigoni et al., 2012; Budd et al., 2012). In recent years, based on the molecular phylogenies by Fukami et al. (2008) and Huang et al. (2011), Merulinidae was redefined and expanded to include all members of ‘Bigmessidae’; Faviidae Gregory, 1900 was demoted to a subfamily (Faviinae) of the new family Faviidae Milne Edwards & Haime, 1957. Meanwhile, Pectiniidae and Trachyphylliidae were regarded as junior synonyms of the family Merulinidae (Huang et al., 2011; Budd et al., 2012).

Members of Merulinidae—namely, *Dipsastraea* de Blainville, 1830, *Favites* Link, 1807, and *Hydnophora* Fischer von Waldheim, 1807—have been closely associated in the past. *Dipsastraea* had never been applied since it was established. Until the recent revision, *Dipsastraea* was redefined to include the Indo-Pacific lineages of *Favia* and all species of *Barabattoia* (now a junior synonym of *Dipsastraea*) (Budd et al., 2012; Huang et al., 2014a). *Favites* is a controversial genus because *Favites pentagona* (Esper, 1795) is more closely related to species of other genera, indicating that *Favites* is polyphyletic (Huang et al., 2011; Huang et al., 2014a; Huang et al., 2014b; Huang et al., 2016; Arrigoni et al., 2012). Huang et al. (2011); Huang et al. (2014a)) discovered that *F. pentagona* clustered more closely with species of *Caulastraea*, *Oulophyllia* and *Pectinia*, than with other *Favites* species based on three nuclear and two mitochondrial loci (28S rDNA, histone H3, ITS rDNA, mt COI and mt IGR). Huang et al. (2014b) and Arrigoni et al. (2012) showed similar results based on histone H3 and COI/ITS genes. *F. pentagona*’s morphology, however, is consistent with other *Favites* species. *Hydnophora* is a distinct genus, and molecular data have supported its monophyly (Huang et al., 2011; Huang, 2012). All three genera are part of the family Merulinidae, but the current interpretations of phylogenetic relationships between *Dipsastraea*, *Favites*, and *Hydnophora* conflict. For instance, Huang et al. (2014a) concluded that *Hydnophora* and *Favites* (with the exception of *F. pentagona*) were more closely related to each other than to *Dipsastraea* species. Huang et al. (2014b), however, found that *Hydnophora*, *F. pentagona*, and subclade B (including *Coelastrea*, *Dipsastraea* and *Trachyphyllia geoffroyi*) were closely related; other *Favites* species formed a separate subclade F. A maximum likelihood genus-level phylogeny (576 species) of Scleractinia based on 12 DNA markers suggested that *Hydnophora* was more closely related to *Dipsastraea* than to *Favites* (Kitahara et al., 2016). More work is needed to clarify the evolutionary relationships between *Dipsastraea*, *Favites*, and *Hydnophora*.

In recent decades, mitochondrial DNA (mtDNA) has frequently been used in phylogeny and molecular evolution studies (Boore, 1999; Curole & Kocher, 1999). Mitochondrion is an important eukaryotic organelle, the mitochondrial genome has become highly economized. It typically includes 13 oxidative phosphorylation (OXPHOS) related genes, two rRNAs that encode the two subunits of mitochondrial ribosomes, and an array of tRNAs used for translation within the organelle. Previous studies revealed that the evolutionary rate of Scleractinia’s mitochondrial genome is 10–20 times slower than that of other metazoan taxa, and 5 times slower than that of its nuclear genome (Van Oppen et al., 2000; Chen et al., 2009). Mitochondrial genome rearrangements occur relatively infrequently within Scleractinia. The mitochondrial genome, therefore, played a significant role in scleractinian studies on phylogeny reconstruction (Fukami & Knowlton, 2005; Arrigoni et al., 2016; Capel et al., 2016; Terraneo et al., 2016a; Terraneo et al., 2016b). It could help us explore Scleractinia’s evolutionary process and clarify the evolutionary relationship between Scleractinia and other Hexacorallia members, such as Actiniaria, Antipatharia, and Corallimorpharia (Fukami & Knowlton, 2005; Kitahara et al., 2014; Lin et al., 2014).

In this study, we sequenced the complete mitochondrial genomes of three corals, *Dipsastraea rotumana*, *Favites pentagona*, and *Hydnophora exesa*, using a next-generation sequencing (NGS) strategy. Moreover, we reexamined the phylogeny of Scleractinia based on 86 species across 15 families using the mitochondrial genomes obtained in this study and those available in GenBank. We aimed to (1) describe and compare the mitogenomes of these corals and (2) provide new perspectives on the phylogenetic relationships within the family Merulinidae. The information obtained in this study may facilitate future phylogenetic and molecular evolutionary studies of Scleractinia.

## Materials & Methods

### Sample collection and DNA extraction

Wild specimens of three Merulinidae species (*D. rotumana*, *F. pentagona*, and *H. exesa*) were collected from Daya Bay (22.56 N, 114.65 E; under 5.2 m), Guangdong Province, China, on 23 November 2015. Each species has only one specimen (Figs. S1–S3). A dissecting microscope was used to identify all specimens based on skeletal morphology characteristics, including the number of septa and denticles, calices and the dimension of calices, in accordance with published taxonomic descriptions (Veron, 2000; Chan et al., 2005). Total genomic DNA was kept at 4 °C after extraction by the DNeasy Tissue Kit (Qiagen, Shanghai, China). DNA concentration was measured by the Nucleic Acid Protein Analyzer (Quawell Technology Inc., Sunnyvale, USA). The genomic DNA extracted with each system was quantified in duplicates with the NanoDrop 2000 spectrophotometer (Thermo Scientific, USA). Additionally, each DNA sample was quantified in duplicates with the Qubit 2.0 fluorometer (Life Technologies, USA).

### Genome sequencing

After the quality control steps, a total of 2 µg double stranded DNA (dsDNA) was sheared to ∼550 bp by the M220 focused ultrasonicator (Covaris, Woburn, MA, USA). Using an Agilent Bioanalyzer 2100 (Agilent Technologies, Santa Clara, CA, USA), fragmented DNA was tested for size distribution, and the library for MiSeq was generated using a TruSeq DNA PCR-free LT Sample Preparation Kit (Illumina, San Diego, CA, USA) according to the manufacturer’s instructions. The final library concentration was determined by real-time quantitative PCR with Illumina adapter-specific primers provided by the KAPA Library Quantification Kit (KAPA Biosystems, Wilmington, MA, USA). Our strategy for assembling the complete mitogenomes was identical to that of Niu et al. (2016). Raw reads were assembled de novo using commercial software (Geneious V9, Auckland, New Zealand) to produce a single, circular complete mitogenome.

### Mitogenome annotation and analyses

DOGMA (Wyman, Jansen & Boore, 2004) and MITOS (Bernt et al., 2013) were used for preliminary annotation, and then protein-coding genes (PCGs) and rRNA genes were annotated by aligning the homologous genes of other reported scleractinian mitogenomes. We also identified and annotated the PCGs and rRNA genes by BLAST searches on the National Center for Biotechnology Information website. Transfer RNA genes were identified by comparing the results predicted by ARWEN, and then the cloverleaf secondary structures of tRNA genes were predicted by tRNAscan-SE 2.0 (Laslett & Canback, 2008; Lowe & Chan, 2016). Codon usage and nucleotide frequencies were calculated by MEGA 6.0 (Tamura et al., 2013). Nucleotide composition skew analysis was carried out with the formulas AT-skew = [A-T] / [A+T] and GC-skew = [G-C] / [G+C] (Perna & Kocher, 1995). We determined the codon usage of all PCGs and used MEGA 6.0 to calculate the Relative Synonymous Codon Usage (RSCU). The rates of nonsynonymous substitutions (Ka) and synonymous substitutions (Ks) for each protein-coding gene were determined with DnaSP 5.0 (Librado & Rozas, 2009).

### Phylogeny reconstruction

We constructed the phylogenetic topology of 86 Scleractinia species using the 13 tandem mitogenome PCG sequences (excluding the stop codon), with 10 Corallimorpharia species as out-groups (Table S1). All sequences obtained in this study were submitted to GenBank. A best-fitting model matrix (Table S2) was chosen by a comparison of the Akaike information criterion (AIC) strategy with jModelTest 2 (Darriba et al., 2012). Based on Bayesian inference (BI) methods, we performed a comprehensive phylogenetic analysis using MrBayes 3.12 (Huelsenbeck & Ronquist, 2001). According to Markov chain Monte Carlo analysis, four chains (one cold and three heated chains) were set to run simultaneously for 1,000,000 generations. Each set was sampled every 100 generations with a burn-in of 25%, and the remaining samples were used to obtain the 50% majority-rule consensus tree.

## Results & Discussion

### Genome organization and composition

**Figure 1:**
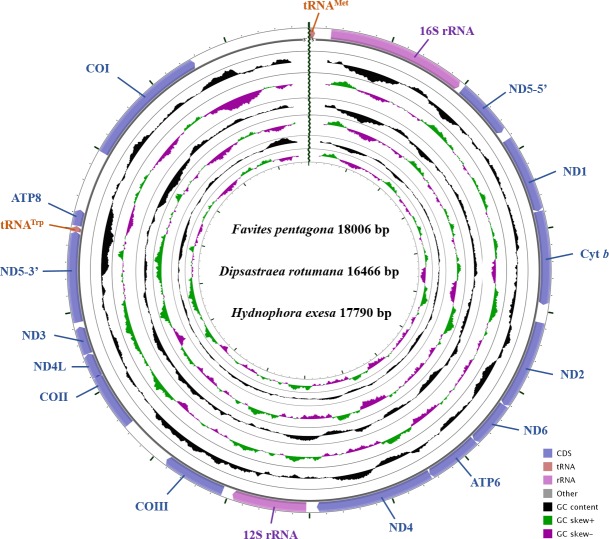
Gene map of the complete mitochondrial genomes for *Dipsastraea rotumana* (MH119077), *Favites pentagona* (KY247139), and *Hydnophora exesa* (MH086217). The larger ring indicates gene arrangement and distribution, the smaller ring indicates the GC content. ND1-6: NADH dehydrogenase subunits 1-6; COI-III: cytochrome c oxidase subunits 1-3; ATP6 and ATP8: ATPase subunits 6 and 8; Cyt *b*: cytochrome b.

The complete mitochondrial genomes of *D. rotumana* (GenBank accession no. MH119077), *F. pentagona* (KY247139), and *H. exesa* (MH086217) were 16,466 bp, 18,006 bp, and 17,790 bp in length, respectively. They carried the typical composition, including 13 PCGs, two transfer RNA genes (tRNA^Met^, tRNA^Trp^) and two ribosomal RNA genes (Fig. 1, Table 1). Length differences were primarily the result of variation in intergenic nucleotides. As found in other Scleractinia species, all PCGs, tRNA, and rRNA genes were encoded on the H-strand. The mitochondrial genome of these three corals were identical to most published scleractinian mitogenomes (Van Oppen et al., 2000; Chen et al., 2008; Wang et al., 2013).

**Table 1:** Summary of gene/element feature of *Dipsastraea rotumana*, *Favites pentagona*, and *Hydnophora exesa*.

**Gene/ Element**	**Strand**	**Size (bp)**	**GC-Percent (%)**	**Amino Acids (aa)**	**InferredInitiation Codon**	**InferredTermination Codon**	**Anticodon**	Intergenic Nucleotide^*^ (bp)
tRNA^Met^	H	72	43.06–44.44				UAC (Y)	674–1448
16S rRNA	H	1698–1951	31.08–31.27					0–193
ND5 5′	H	711	32.35–32.84	236	ATG			0–49
ND1	H	948	34.81–35.17	315	ATG	TAG		109–110
Cyt *b*	H	1140	33.33–33.77	379	ATG	TAA		2
ND2	H	1287	31.88–32.42	428	ATA	TAA		25
ND6	H	561	30.84–31.55	186	ATG	TAA		1
ATP6	H	678	32.45–32.68	225	ATG	TAA		−1
ND4	H	1440	32.24–33.82	479	ATG	TAG		−1
12S rRNA	H	910–911	34.58–35.05					108–138
COIII	H	780	36.79–37.18	259	GTG	TAA		67–125
COII	H	708	33.63–34.6	235	ATG	TAA		626–713
ND4L	H	300	28.67–29.96	99	ATG	TAA		–19
ND3	H	342	31.29–31.76	113	ATG	TAA		2
ND5 3′	H	1104	30.34–30.53	367		TAG		56
tRNA^Trp^	H	70–71	39.44–40.00				AUC (I)	−2
ATP8	H	198	19.70–20.71	65	ATG	TAA		3
COI	H	1590	35.43–35.94	529	ATG	TAA		−1–724

* As for the adjacent genes, positive number represented spaced, negative number indicated overlap.

### Nucleotide composition

The overall nucleotide compositions of the three corals in descending order were 41.6% T, 25.2% A, 20.3% G, and 12.9% C for *D. rotumana*; 41.1% T, 25.3% A, 20.2% G, and 13.3% C for *F. pentagona*; 41.7% T, 25.0% A, 20.4% G, and 13.0% C for *H. exesa*; respectively. The dominant base of protein-coding genes was T and the dominant base of tRNA genes and rRNA genes was A. C was the least common base in all regions of the mitogenome for all three coral species, as is common in most species of Scleractinia (Fig. S4, Table 2). The nucleotide compositions of 14 related Scleractinia mitogenomes were also presented (Table 2). AT-skews analysis among these 14 species were all negative, while their GC-skews were all positive for both entire mitogenomes and protein-coding genes. AT-skew and GC-skew analyses indicated that in the complete mitogenomes, bases A and T were favored, whereas C was not. This was consistent with previous observations of most Scleractinia speices (Brugler & France, 2007; Niu et al., 2016). Regarding the PCGs, the ND3 gene showed the smallest value for AT-skews. The ATP8 gene showed the highest. The ND3 gene showed the highest value for GC-skews, whereas the ATP8 gene showed the lowest. *H. exesa*’s ATP8 gene had a negative value (Fig. S5).

**Table 2:** Summary of the base composition of the mitogenomes at each codon position of the concatenated 13 protein-coding genes (PCGs) across *Dipsastraea rotumana*, *Favites pentagona*, *Hydnophora exesa* and other 11 related species of Scleractinia.

**Species**	**Accessionnumber**	**Length(bp)**	**Entire genome**						**Protein-coding gene**			
			**A (%)**	**T (%)**	**C (%)**	**G (%)**	**AT-Skew**	**GC-Skew**	**Length (aa)**	**AT (%)**	**AT-Skew**	**GC-Skew**
*Astrangia poculata*	NC_008161	14,853	25.2	42.9	12.2	19.7	−0.259	0.233	3837	68.039	−0.345	0.231
*Colpophyllia natans*	NC_008162	16,906	24.9	41.5	13.2	20.3	−0.250	0.211	3847	67.040	−0.351	0.224
*Cyphastrea serailia*	KY094484	17,138	25.0	41.4	13.0	20.5	−0.247	0.225	3916	66.735	−0.350	0.219
*Echinophyllia aspera*	MG792550	17,697	25.3	40.6	13.4	20.7	−0.231	0.212	3943	66.044	−0.349	0.211
*Favites abdita*	NC_035879	17,825	25.0	41.2	13.3	20.5	−0.245	0.213	3837	66.562	−0.349	0.217
***Favites pentagona***	KY247139	18,006	25.3	41.1	13.3	20.2	−0.238	0.206	3915	66.502	−0.349	0.216
***Dipsastraea rotumana***	MH119077	16,466	25.2	41.6	12.9	20.3	−0.246	0.221	3915	66.769	−0.351	0.223
***Hydnophora exesa***	MH086217	17,790	25.0	41.7	13.0	20.4	−0.251	0.224	3915	66.863	−0.350	0.223
*Mussa angulosa*	NC_008163	17,245	25.1	41.2	13.4	20.3	−0.242	0.203	3850	66.814	−0.349	0.220
*Orbicella annularis*	NC_007224	16,138	24.9	41.5	13.1	20.4	−0.251	0.217	3912	66.385	−0.352	0.220
*Orbicella faveolata*	NC_007226	16,138	24.9	41.5	13.2	20.4	−0.250	0.217	3912	66.377	−0.352	0.220
*Orbicella franksi*	NC_007225	16,137	24.9	41.5	13.2	20.4	−0.250	0.215	3912	66.402	−0.351	0.219
*Platygyra carnosa*	NC_020049	16,463	25.6	41.4	12.8	20.1	−0.236	0.222	3916	66.658	−0.349	0.221
*Sclerophyllia maxima*	FO904931	18,168	25.3	41.0	13.1	20.6	−0.237	0.221	3943	66.863	−0.354	0.231

### Protein-coding genes

It is worth noting that the mitogenomes of all three Merulinidae species showed an intron insertion in the protein-coding gene ND5 (positions 10,147–10,243). The ND5 intron contained ten protein-coding genes and one rRNA gene, which was consistent with the canonical sequence in scleractinian mitogenomes (Type SII Lin et al., 2014). In the mitogenomes of *D. rotumana*, *F. pentagona*, and *H. exesa*, the COIII gene started with GTG, the ND2 gene started with ATA, and all of the remaining protein-coding genes used ATG as the start codon. In addition, three of the 13 PCGs (ND1, ND4, and ND5) used TAG as the stop codon, and the other ten PCGs (Cyt *b*, ATP6, ND2, ND4L, ND3, ND6, COIII, COII, COI, and ATP8) used TAA as the stop codon.

### Mitochondrial gene codon usage

**Figure 2:**
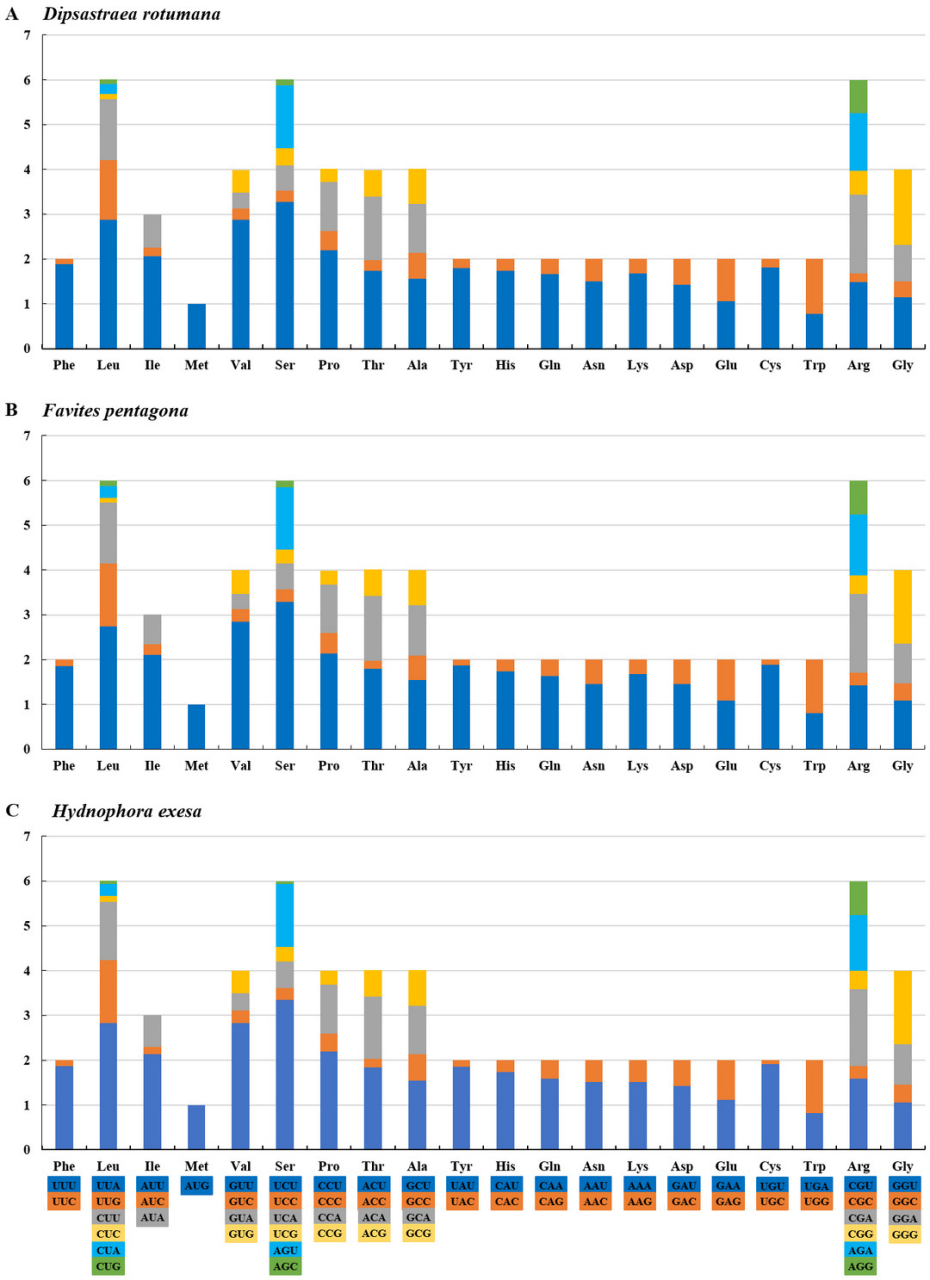
RSCU (Relative Synonymous Codon Usage) of mitochondrial genomes for (A) *Dipsastraea rotumana*, (B) *Favites pentagona*, and (C) *Hydnophora exesa*.

**Figure 3:**
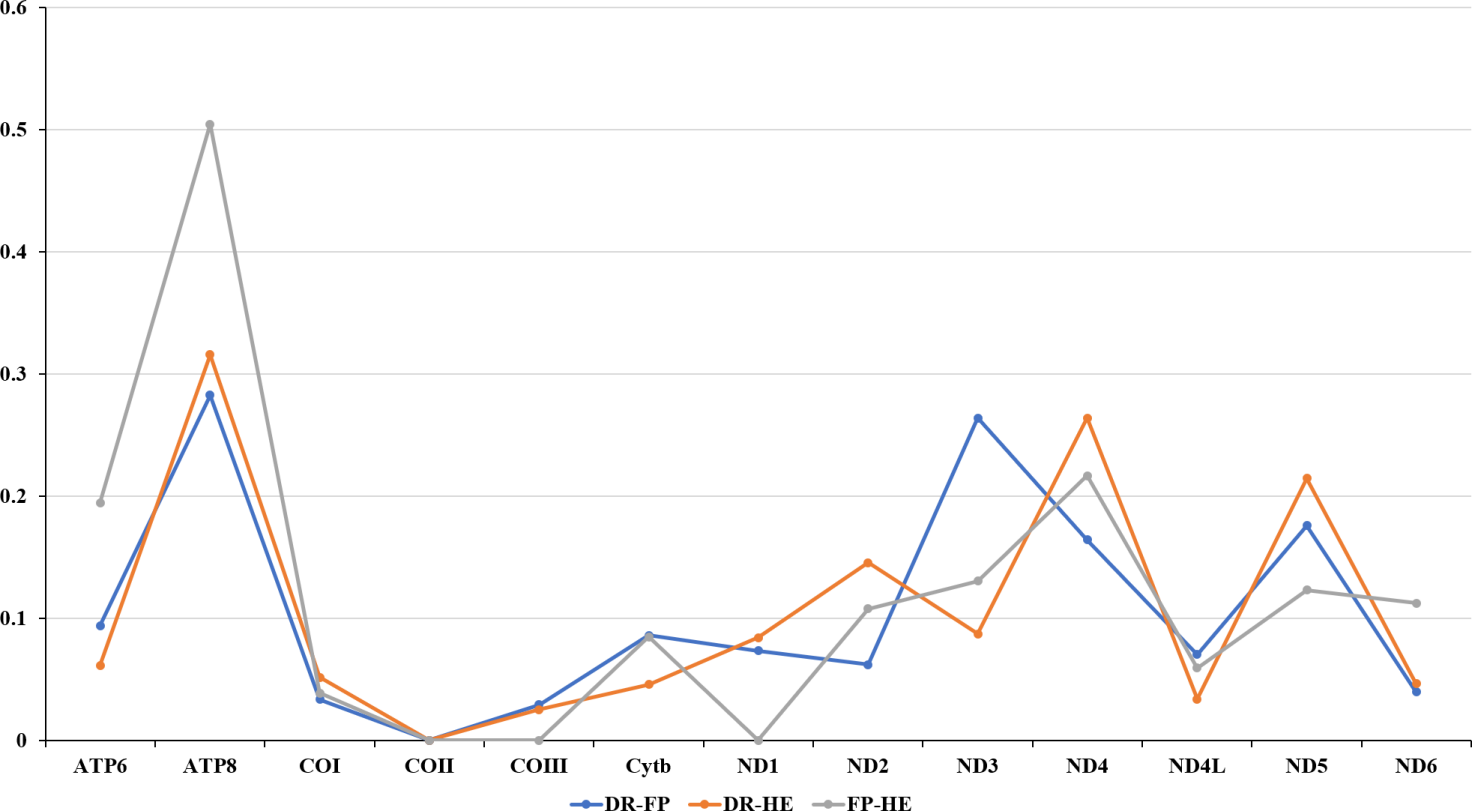
Evolutionary rates of the mitochondrial genome of *Dipsastraea rotumana* (DR), *Favites pentagona* (FP), and *Hydnophora exesa* (HE). The ratio means the rate of non-synonymous substitutions to the rate of synonymous substitutions (Ka/Ks) for each PCG.

The amino acids Leu, Ser, and Arg, which were encoded by six different codons, appeared more frequently than other amino acids (Fig. 2). For all 13 mitochondrial PCGs of *D. rotumana*, *F. pentagona*, and *H. exesa*, the nonsynonymous/synonymous mutation ratio (Ka/Ks) varied from 0 to 0.5043 (Fig. 3). Nonsynonymous substitutions are generally more harmful than synonymous substitutions. In mitogenomes, some genes may play more important roles than other genes. Therefore, it is reasonable to assume that in order to maintain their function some genes have undergone stronger selective constraints to eliminate deleterious mutations than others. The analysis of the Ka/Ks ratios indicated that the mitochondrial PCGs evolved under strong purifying selection, which signified natural selection against deleterious mutations with negative selection coefficients (Yang & Bielawski, 2000). The Ka/Ks ratios varied for different genes, implying that different genes accumulated different amounts of deleterious mutations. The results showed that ATP8 genes in 13 PCGs presented the highest Ka/Ks values, indicating that the ATP8 gene was under minor selection pressures (Lynch, Koskella & Schaack, 2006).

### Ribosomal and transfer RNA genes

As is the case in most Scleractinia mitogenomes, two tRNA genes (tRNA-met and tRNA-trp) were found in the mitogenomes of *D. rotumana*, *F. pentagona*, and *H. exesa*. All three species exhibited a tRNA-met composed of 72 bp, yet the tRNA-trp gene varied in length from 70 bp (*D. rotumana*) to 71 bp (*H. exesa* and *F. pentagona*), with some subtle base composition difference between the species. Both tRNAs carried identical anticodons, as reported in other Scleractinia species (Niu et al., 2016; Terraneo et al., 2016a; Terraneo et al., 2016b). In addition, both tRNA genes were folded into typical cloverleaf secondary structures (Fig. S6), containing an amino acid acceptor stem, T *ψ*C stem, anticodon stem, and DHU stem. Two rRNAs genes ranged in length from 910 to 911 bp (12S rRNA) and from 1698 to 1,699 bp (16s rRNA), respectively.

### Phylogenetic analyses

**Figure 4:**
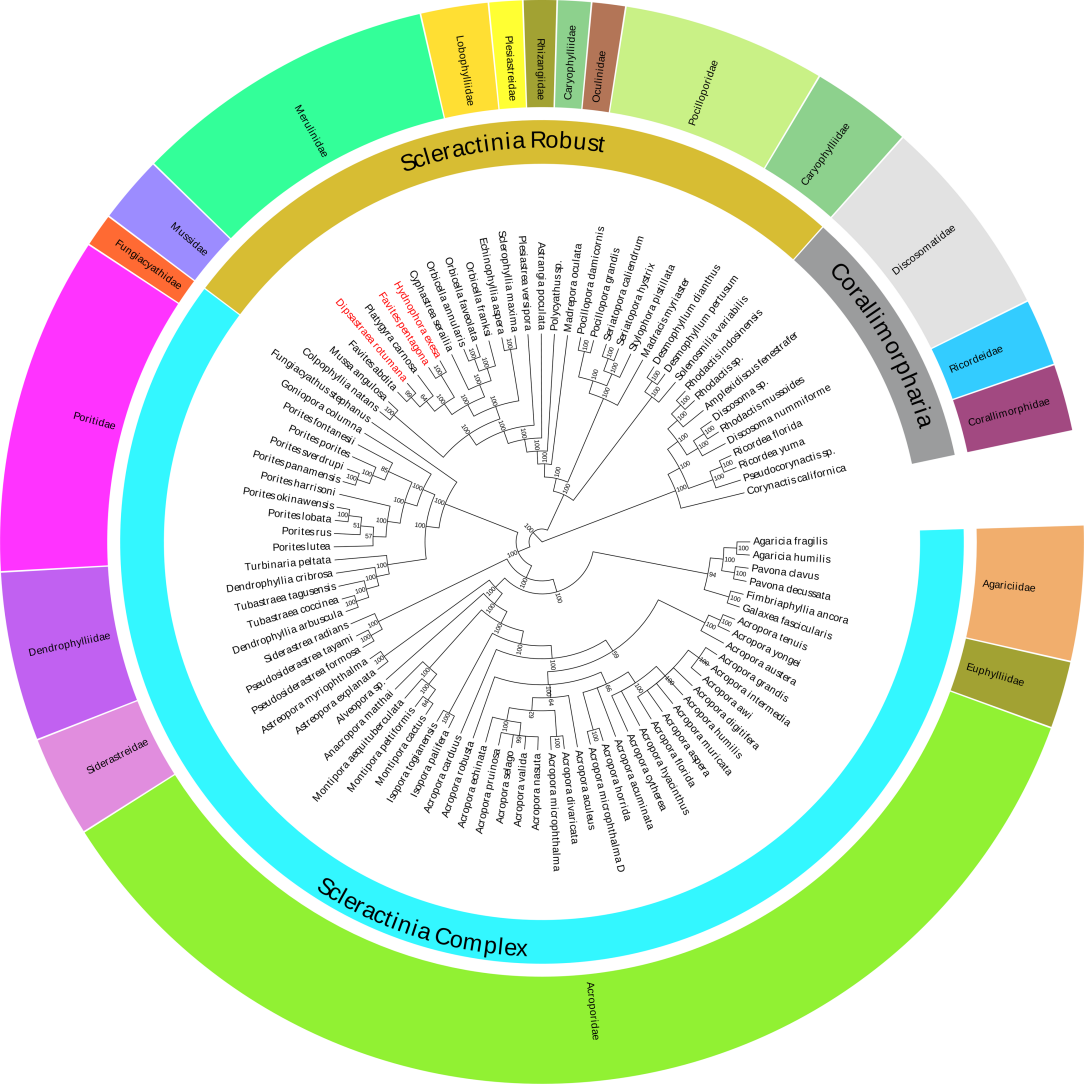
Bayesian inference (BI) tree inferred from the amino acid sequences of 13 PCGs of 86 Scleractinia species and 10 Corallimorpharia species as out-groups. The numbers at the nodes showed the Bayesian posterior probabilities. The species in red Latin name indicated the sequences generated in this study.

In this study, the Bayesian analysis was constructed based on the 13 concatenated PCG sequences of 86 Scleractinia species (Table S1). The Bayesian phylogenetic tree indicated that all of the interspecific nodes were robust, with strong posterior probabilities (Fig. 4). Species in the same family were grouped together, with the exception of the *Polycyathus* sp. (NC_015642), which was distinct from other Caryophylliidae species. The family Merulinidae was conventionally clustered into a robust branch and affined with the family Lobophylliidae. Unexpectedly, based on the mitochondrial genome phylogeny, the topological structure of the relationship between *H. exesa*, *F. pentagona*, *D. rotumana*, and *F. abdita*, was not consistent with previous phylogenies (Huang et al., 2011; Huang et al., 2014a; Huang et al., 2014b; Huang et al., 2016; Katahara et al., 2016). For example, Huang et al. (2014a) found that *F. pentagona* and *D. rotumana* shared a close evolutionary relationship, whereas *H. exesa* clustered more closely with other *Favites* species according to three nuclear and two mitochondrial markers. Almost all previous molecular studies rendered the genus *Favites* as polyphyletic. As described above, we also found that, the genus *Favites* was polyphyletic, with *F. pentagona* clustered closely with *H. exesa*, and *D. rotumana* clustered closely with *F. abdita*. These results indicate novel interspecific relationships.

## Conclusions

In the present study, the complete mitochondrial genome sequences of three Merulinidae corals (*D. rotumana*, *F. pentagona*, and *H. exesa*) were determined for the first time using next-generation sequencing. The gene arrangement and composition of the three corals were identical to each other and consistent with other Scleractinia mitogenomes. The Bayesian inference results showed that the family Merulinidae was clustered into the robust branch, with *H. exesa* clustered closely with *F. pentagona* and *D. rotumana* clustered closely with *F. abdita*. This study provides reliable mitogenome data and novel insight into the phylogenetic relationships of the genera in the family Merulinidae. Our study may also facilitates future phylogenetic and evolutionary studies of Scleractinia.

##  Supplemental Information

10.7717/peerj.8455/supp-1 Dataset S1 Raw data of mt-genome sequence of *D. rotumana.* The complete mitochondrial genome of *D. rotumana* was 16,466 bp in length with GenBank accession no. MH119077.

10.7717/peerj.8455/supp-2 Dataset S2 Raw data of mt-genome sequence of *F. pentagona.* The complete mitochondrial genome of *F. pentagona* was 18,006 bp in length with GenBank accession no. KY247139.

10.7717/peerj.8455/supp-3 Dataset S3 Raw data of mt-genome sequence of *H. exesa.* The complete mitochondrial genome of *H. exesa* was 17,790 bp in length with GenBank accession no. MH086217.

10.7717/peerj.8455/supp-4 Figure S1 Underwater picture of *Dipsastraea rotumana* Corallites are large, with diameter over 12 mm; deep and subplocoid, with a cerioid tendency especially towards the top surfaces of the coral. Corallites can appear completely cerioid, highly packed and crowded. Corallite shapes are irregular and vary within a single colony. Septa are very exsert and have permanent dentation of irregular length.

10.7717/peerj.8455/supp-5 Figure S2 Underwater picture of *Favites pentagona* Corallites are typically cerioid, angular-shaped with thin walls, i.e., honeycomb shapes, with small size of about 5–6 mm in diameter. Corallite walls have a distinctive demarcations of light grey or white, with dark brown or grey inner walls.

10.7717/peerj.8455/supp-6 Figure S3 Underwater picture of *Hydnophora exesa* Unique skeletal structures, with highly pointed small conical mounds, growing throughout the colony surface. They can reach up to 8–10 mm in diameter. Septa are irregular and vary within a single colony.

10.7717/peerj.8455/supp-7 Figure S4 Codon usage bias in different regions of mitochondrial genome of *Dipsastraea rotumana*, *Favites pentagona*, and *Hydnophora exesa* PCGs, protein coding genes; 1st, the first positions of codons; 2nd, the second positions of codons; 3rd, the third positions of codons.

10.7717/peerj.8455/supp-8 Figure S5 Graphical illustration showing the AT- and GC-skew in the PCGs of the mitochondrial genome of (A) *Dipsastraea rotumana*, (B) *Favites pentagona*, and (C) *Hydnophora exesa*

10.7717/peerj.8455/supp-9 Figure S6 Secondary structures of *Dipsastraea rotumana* tRNAs Single variable sites from two species are labelled in different colours (*Favites pentagona*: red; *Hydnophora exesa*: green).

10.7717/peerj.8455/supp-10 Table S1 96 species and their taxonomy of series corals for the phylogenetic tree

10.7717/peerj.8455/supp-11 Table S2 A best-fitting model matrix for 13 PCGs

10.7717/peerj.8455/supp-12 Supplemental Information 12 Raw data of mt-genome sequence of *F. pentagona* D36*.*

10.7717/peerj.8455/supp-13 Supplemental Information 13 Raw data of 28S gene sequence of *F. pentagona* D36*.*

10.7717/peerj.8455/supp-14 Supplemental Information 14 Raw data of histone H3 gene sequence of *F. pentagona* D36

## Additional Information and Declarations

### Data Availability

The following information was supplied regarding data availability: The complete mitochondrial sequences are available at GenBank: MH119077 for *D. rotumana*, KY247139 for *F. pentagona*, and MH086217 for *H. exesa*.
