# Supplementary figures and images for: Characterization of the complete mitochondrial genome sequences of three Merulinidae corals and novel insights into the phylogenetics
Source: PeerJ. 2020 Jan 24;8:e8455. doi: 10.7717/peerj.8455 (PMC6984341; doi:10.7717/peerj.8455)

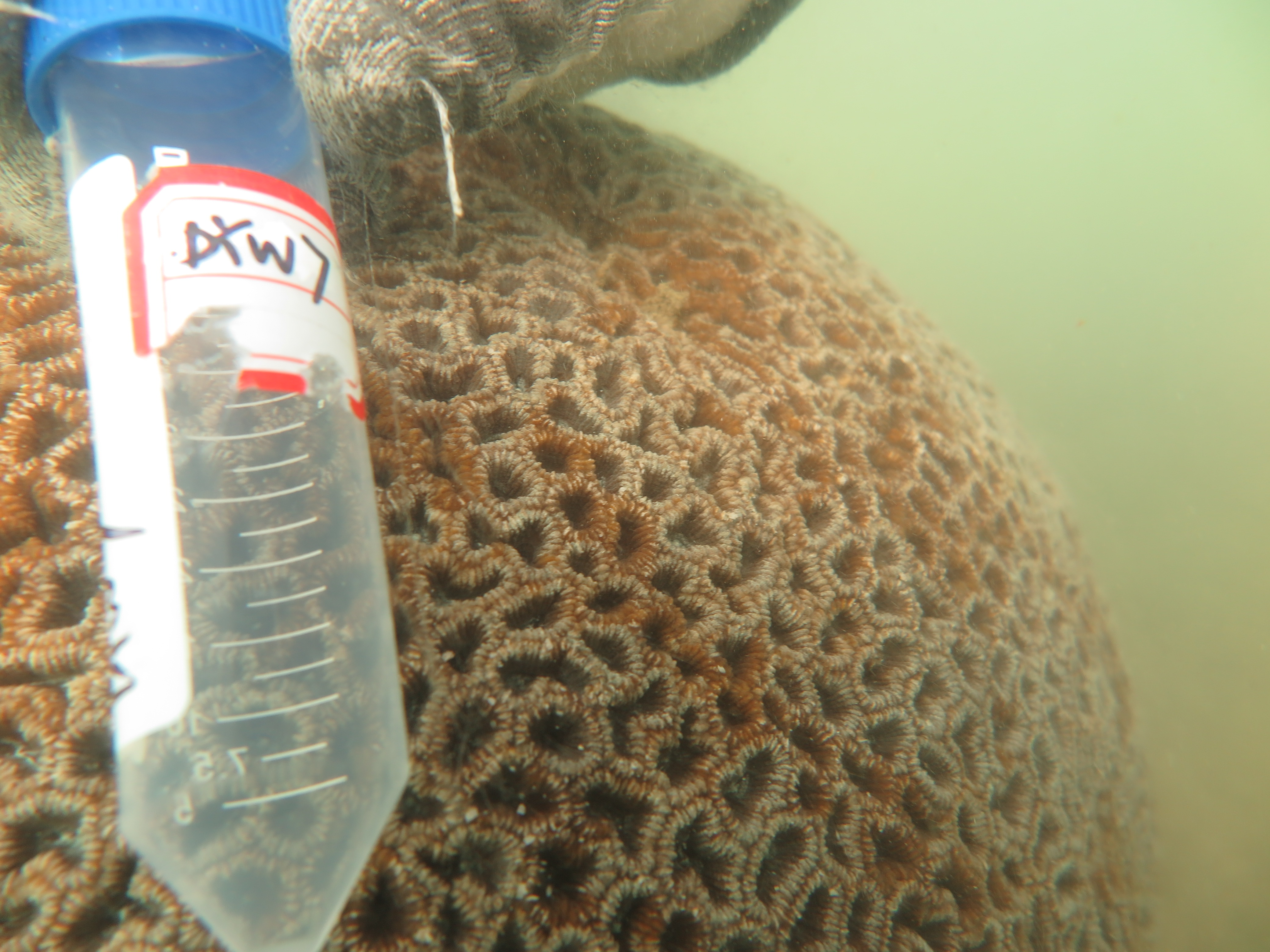

Supplement: Figure S1 — Corallites are large, with diameter over 12 mm; deep and subplocoid, with a cerioid tendency especially towards the top surfaces of the coral. Corallites can appear completely cerioid, highly packed and crowded. Corallite shapes are irregular and vary within a single colony. Septa are very exsert and have permanent dentation of irregular length. [file peerj-08-8455-s004.png]

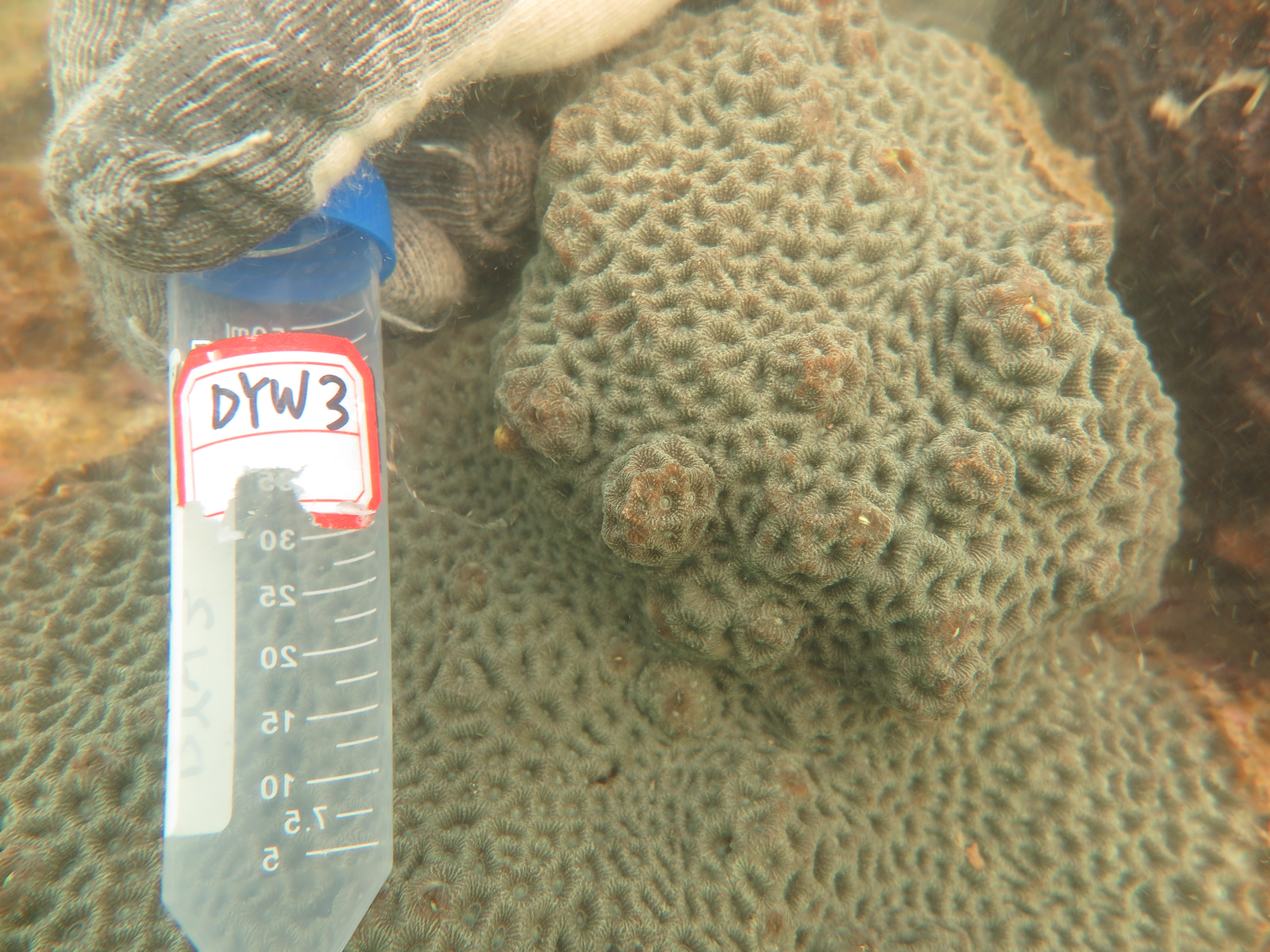

Supplement: Figure S2 — Corallites are typically cerioid, angular-shaped with thin walls, i.e., honeycomb shapes, with small size of about 5–6 mm in diameter. Corallite walls have a distinctive demarcations of light grey or white, with dark brown or grey inner walls. [file peerj-08-8455-s005.png]

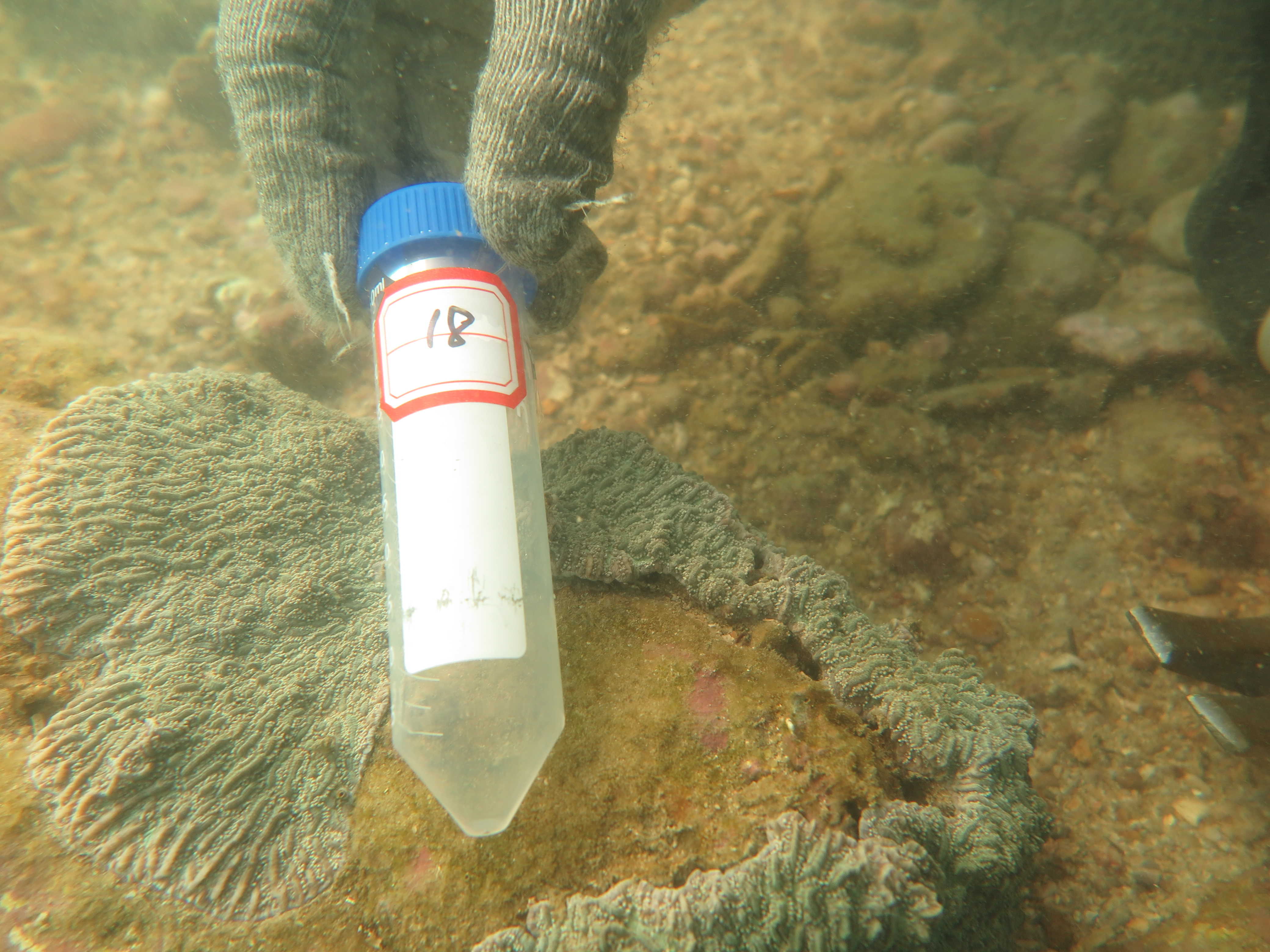

Supplement: Figure S3 — Unique skeletal structures, with highly pointed small conical mounds, growing throughout the colony surface. They can reach up to 8–10 mm in diameter. Septa are irregular and vary within a single colony. [file peerj-08-8455-s006.png]

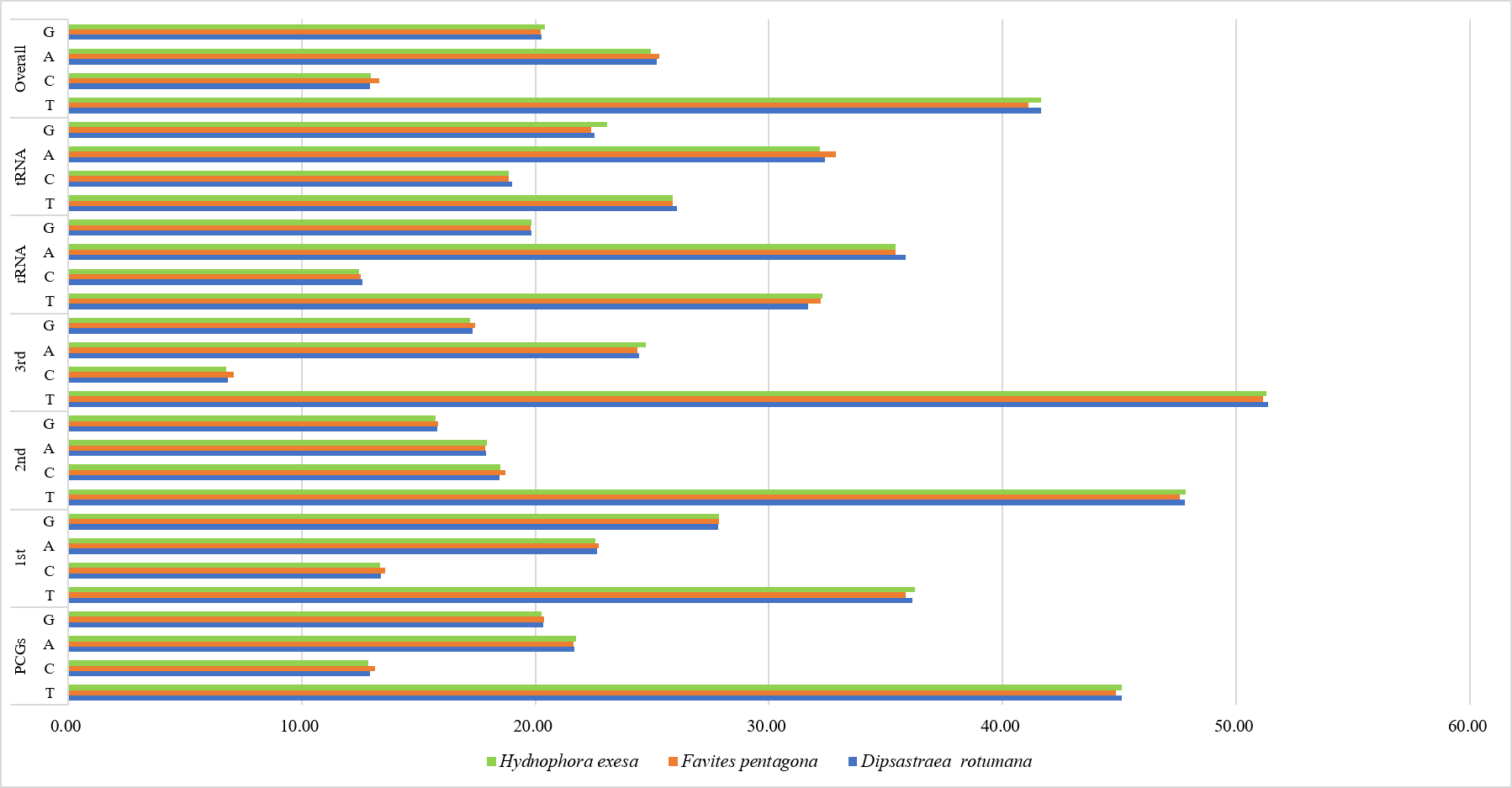

Supplement: Figure S4 — PCGs, protein coding genes; 1st, the first positions of codons; 2nd, the second positions of codons; 3rd, the third positions of codons. [file peerj-08-8455-s007.png]

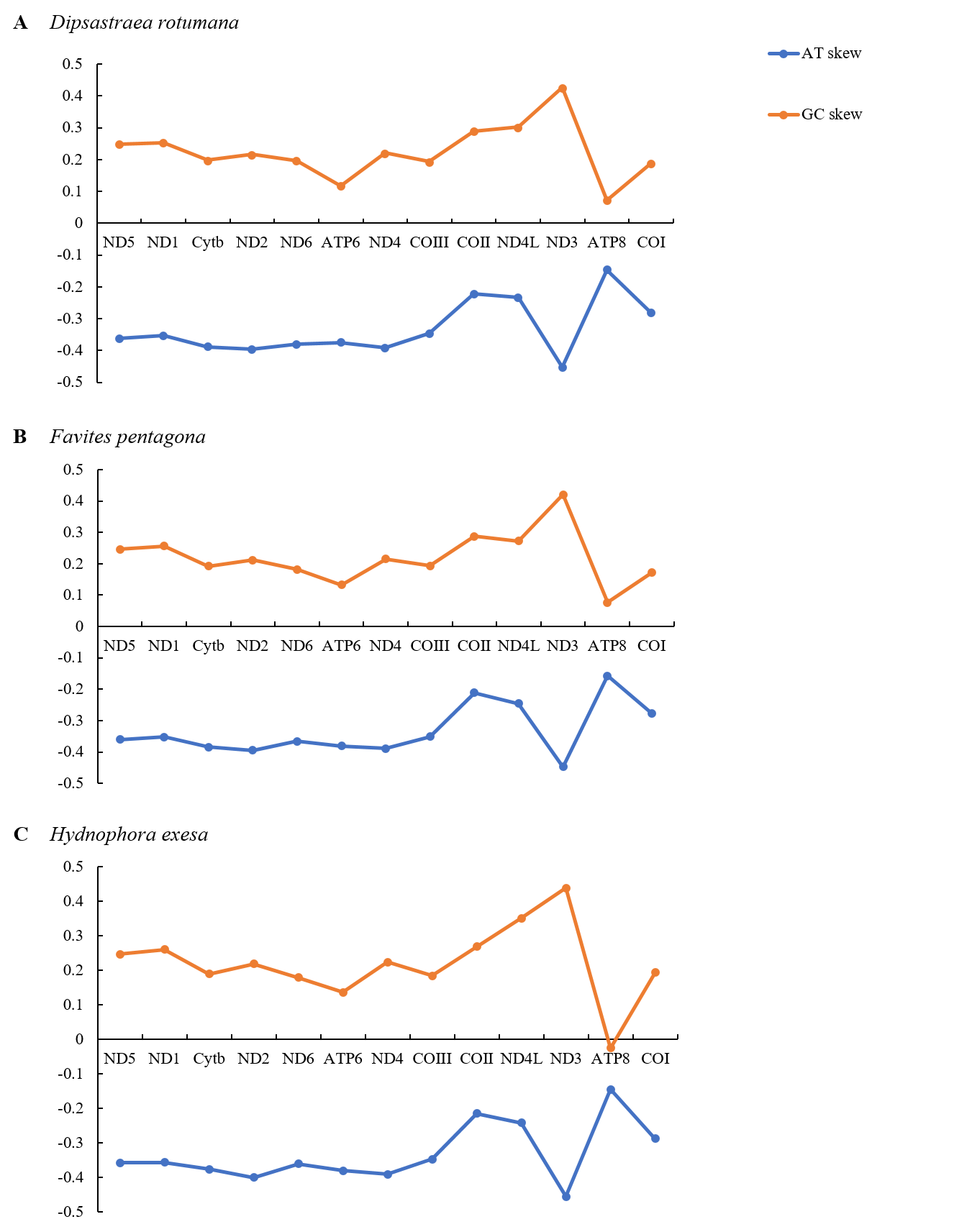

Supplement: Figure S5 [file peerj-08-8455-s008.png]

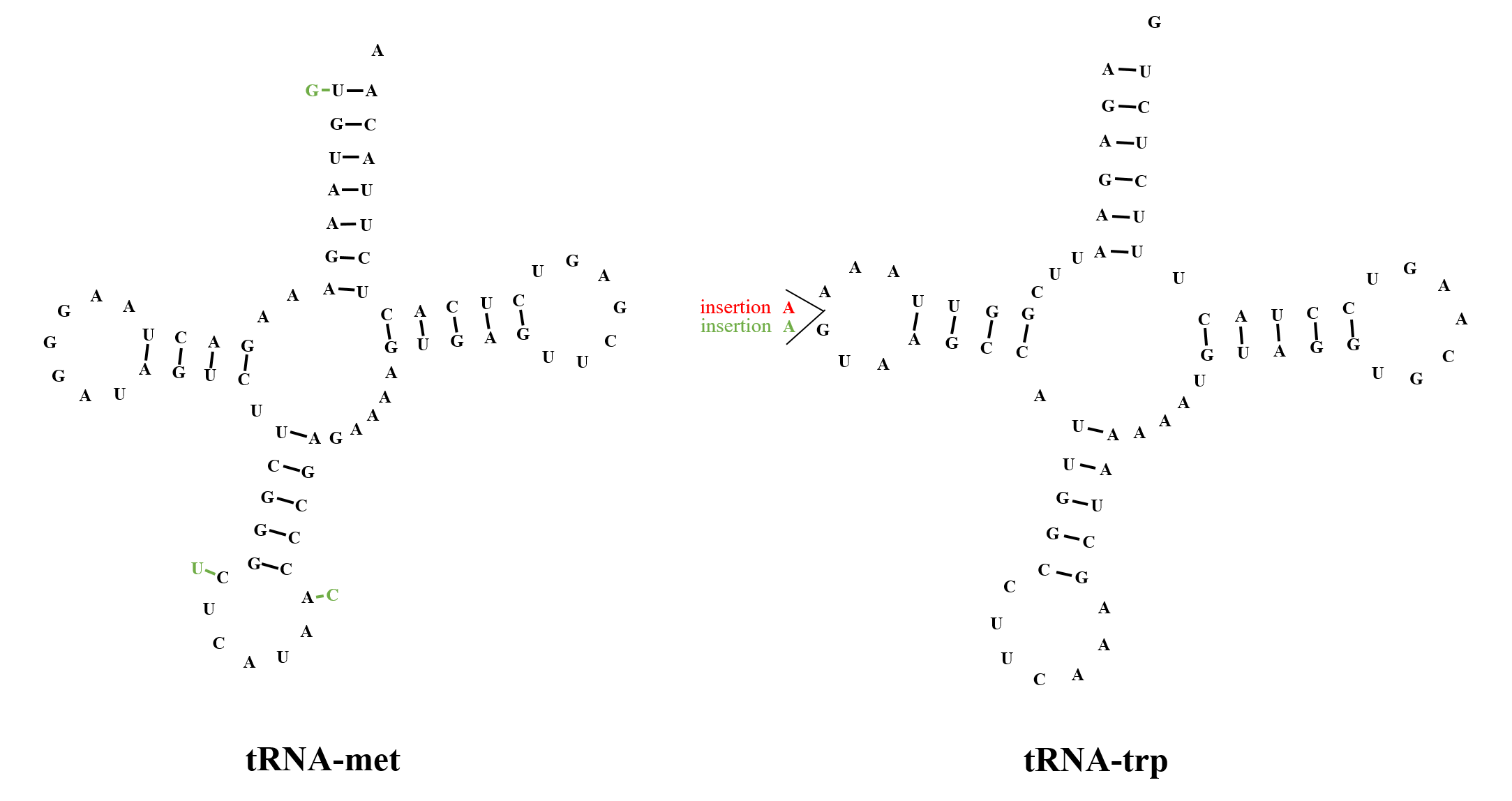

Supplement: Figure S6 — Single variable sites from two species are labelled in different colours (Favites pentagona: red; Hydnophora exesa: green). [file peerj-08-8455-s009.png]
